# Transcriptome analysis of MYB transcription factors family and *PgMYB* genes involved in salt stress resistance in *Panax ginseng*

**DOI:** 10.1186/s12870-022-03871-8

**Published:** 2022-10-08

**Authors:** Mingming Liu, Ke Li, Shichao Sheng, Mingyu Wang, Panpan Hua, Yanfang Wang, Ping Chen, Kangyu Wang, Mingzhu Zhao, Yi Wang, Meiping Zhang

**Affiliations:** 1 College of Life Science, Jilin Agricultural University, Changchun, 130118 Jilin China; 2 Jilin Engineering Research Center Ginseng Genetic Resources Development and Utilization, Changchun, 130118 Jilin China; 3 Laboratory for Cultivation and Breeding of Medicinal Plants of National Administration of Traditional Chinese Medicine, Jilin Agricultural University, Changchun, 130118 Jilin China

**Keywords:** *Panax ginseng*, MYB transcription factor, Phylogeny, Gene expression, Gene interaction network, Salt stress resistance

## Abstract

**Background:**

As the king of all herbs, the medicinal value of ginseng is self-evident. The perennial nature of ginseng causes its quality to be influenced by various factors, one of which is the soil environment. During plant growth and development, MYB transcription factors play an important role in responding to abiotic stresses and regulating the synthesis of secondary metabolites. However, there are relatively few reports on the MYB transcription factor family in *Panax ginseng*.

**Results:**

This study identified 420 *PgMYB* transcripts under 117 genes ID in the Jilin ginseng transcriptome database. Phylogenetic analysis showed that *PgMYB* transcripts in Jilin ginseng were classified into 19 functional subclasses. The GO annotation result indicated that the functional differentiation of *PgMYB* transcripts was annotated to 11 functional nodes at GO Level 2 in ginseng. Expression pattern analysis of *PgMYB* transcripts based on the expression data (TPM) that *PgMYB* transcripts were revealed spatiotemporally specific in expression patterns. We performed a weighted network co-expression network analysis on the expression of *PgMYB* transcripts from different samples. The co-expression network containing 51 *PgMYB* transcripts was formed under a soft threshold of 0.85, revealing the reciprocal relationship of *PgMYB* in ginseng. Treatment of adventitious roots of ginseng with different concentrations of NaCl revealed four up-regulated expression of *PgMY*B transcripts that can candidate genes for salt resistance studies in ginseng.

**Conclusions:**

The present findings provide data resources for the subsequent study of the functions of MYB transcription factor family members in ginseng, and provide an experimental basis for the anti-salt functions of MYB transcription factors in *Panax ginseng*.

**Supplementary Information:**

The online version contains supplementary material available at 10.1186/s12870-022-03871-8.

## Introduction

*Panax ginseng*, a member of the perennial herb genus Ginseng in the family of Araliaceae, has been used medicinally for millennia. As a traditional and valuable Chinese medicine, ginseng is mainly used to treat weakness and fatigue, improve mental function, exercise capacity, immune function, and diabetes-related diseases [1]. As we all know, the dried root of ginseng is the main medicinal part of ginseng, and ginsenoside is the main active ingredient of ginseng. Ginsenosides are found in different tissues such as roots, leaves, stems, flower buds, and berries [2]. Ginsenoside content is affected by the age of growth and is also affected by various conditions such as species and cultivation area [3, 4]. Meanwhile, abiotic stresses can seriously affect the quality of ginseng during the years of growth. Important regulators of transcription factors in plant growth and development and secondary metabolism. According to previous studies, transcription factors such as WRKY [5], MYB [6], NAC [7], and ERF [8] play important roles in plant response to abiotic stresses.

MYB transcription factors, one of the largest family transcription factors in plants [9], were first reported in maize [10]. The MYB structural domain usually consists of one to three incomplete repeats of approximately 52 amino acid residues in a helix-turn-helix conformation inserted into the main DNA groove [11]. The MYB transcription factor family has been divided into subgroups based on the number of DNA-binding domains they contain, such as 1R-MYB, R2R3-MYB, 3R-MYB and 4R-MYB. Interestingly, in recent years, research on R2R3-MYB has been hotly pursued. MYB research has progressed hotly, while 4R-MYB has been less reported. So far, MYB transcription factors have been reported in Arabidopsis [12], rice [13], soybean [14], *Hedychium coronarium* [15], watermelon [16] and other plants have been identified. In rice, OsMYB2 was associated with salt, cold and dehydration tolerance [17] GmMYB12B2 affects the expression level of key enzyme genes for flavonoid biosynthesis in transgenic Arabidopsis [18]. Activating R2R3-myb genes induce repressive R2R3-myb genes to balance anthocyanin and proanthocyanidin accumulation [19] MYB transcription factors play an important role in regulating secondary metabolite biosynthesis, turgor development and stress response in tea tree [20]. In ginseng, the expression of *PgMYB1* was up-regulated by abscisic acid, salicylic acid, NaCl, and cold (chilling), and down-regulated by methyl jasmonate. These results suggest that *PgMYB1* might be involved in responding to environmental stresses and hormones [21]. PgMYB2 protein can bind to the promoter of *PgDDS*, which in turn regulates the expression of ginsenosides [22]. Although there are reports of MYB family members in ginseng, only two ginseng *MYB* genes have been cloned, and they were both cloned using homologous sequences from heterologous species as candidate genes. This study provides a data resource for subsequent studies of the MYB transcriptional family in ginseng and provides candidate genes for ginseng research in salt resistance.

In this study, we identified and analyzed the MYB transcription factor family members in ginseng and obtained 420 *PgMYB* transcripts under 117 gene IDs. We identified the evolutionary relationships and conserved motifs present in *PgMYB* transcripts and uncovered the potential functions of these transcripts. Weighted network co-expression revealed co-expression relationships among *PgMYB* transcripts, suggesting a synergistic effect when they exercise their functions. After treatment of adventitious roots of ginseng with different concentrations of NaCl, the fluorescence quantification results showed that the gene expression of *PgMYB01*, *PgMYB71–01*, *PgMYB71–03* and *PgMYB71–05* were increased to different degrees.

## Materials and methods

### Identification of the *MYB* genes in Jilin ginseng

This study is based on the Jilin ginseng transcriptome database. In order to ensure the integrity of the MYB transcription factor family in Jilin ginseng as much as possible, we took three different approaches in our methodology [23]. First, the seed file of the hidden Markov model containing the MYB_DNA-binding (PF00249) structural domain was downloaded from the PFAM (http://pfam.xfam.org/) protein database, and the protein sequences containing MYB structural domain were retrieved from the Jilin Ginseng Protein Database using native HMMER software (http://hmmer.org/Download.html), and the nucleic acid sequences of Jilin ginseng were compared by tBlastn at 1.0E-06. Second, we downloaded the CDS and protein sequences associated with MYB transcription factors from the Korean Ginseng Genome website (http://ginsengdb.snu.ac.kr/pathway.php), respectively Jilin ginseng transcriptome database to obtain the relevant transcripts. Third, we chose to download 10 sequences of MYB proteins that have been verified to function from the NCBI database, which were from medicago [24], tobacco [25, 26], Arabidopsis [18, 27], strawberry [28], poplar [29], petunia [30], wheat [31], citrus [32]. Similarly, these 10 protein sequences were compared to the Jilin ginseng transcriptome database to obtain the corresponding transcripts. Subsequently, the nucleic acid sequence results obtained from the three comparisons were intersected to remove the duplicate information. Subsequently, the above results were uploaded to iTAK (http://itak.feilab.net/cgi-bin/itak/index.cgi), and finally, the CD Search in NCBI (https://www.ncbi.nlm.nih.gov/Structure/cdd/wrpsb.cgi) for further identification of their structural domains and further validation of the structural domains of PgMYB proteins by SMART (http://smart.embl-heidelberg.de/). Finally, they were named *PgMYB* + numbers (Table S1).

### Structural characteristics and phylogeny of the *PgMYB* transcripts

Through the SMART online tool analysis, we obtained 257 PgMYB transcripts containing the MYB structural domain (Table S2), which were analyzed by the MEGA X [33]. Phylogenetic analysis of these transcripts was performed using the Neighbor-Joining (NJ) method, bootstrap repeats 1000, other settings as default parameters, and finally, they were embellished on iTOL (https://itol.embl.de/). To understand the structure of these transcripts, we predicted their conserved motifs by the online tool MEME (https://meme-suite.org/meme/tools/meme). Also, the contained structural domains of PgMYB transcription factors were analyzed by NCBI CDD tool (https://www.ncbi.nlm.nih.gov/Structure/bwrpsb/bwrpsb.cgi). Finally, they were visualized by TBtools [34] to visualize them.

### *PgMYB* gene duplication and chromosome localization

The 420 *PgMYB* genes from Jilin ginseng were compared locally with ginseng genomic data [35]. The transcript data with 100% match to the chromosome were selected for analysis. The positions of *PgMYB* transcripts on the chromosomes were then visualized by the online tool MG2C V2.1 (http://mg2c.iask.in/mg2c_v2.1/index.html). Subsequently, sequences with ≥99% similarity in the matched regions and transcripts ≥300 bp in length were selected for intra-species covariance analysis, and the circles of *PgMYB* transcripts on chromosomes with duplicated gene pairs were mapped by R language.

### Functional differentiation of the *PgMYB* gene family

The resulting *PgMYB* transcripts were submitted to Blast2GO V6.0.3 [36] for gene ontology annotation. The annotation results were used to classify these transcripts into functions and count the number of transcripts annotated to different functions (Biological Process, Molecular Function, Cellular Component). The functional subclasses of *PgMYB* transcripts annotated under Level 2 were visualized by R language (Table S3).

### Expressions and network analysis of the *PgMYB* transcripts

During variable splicing of genes, different transcripts are formed, and these transcripts form proteins with different potential functions through the process of translation. Therefore, we retrieved the expression data of *PgMYB* transcripts from the expression database of Jilin ginseng transcripts (from 42 farmer’s cultivar roots, 14 different tissues, and four different-year-old ginseng roots) and explored the expression patterns of these *PgMYB* transcripts (Table S4).

### Weight correlation network analysis of the *PgMYB* gene transcripts

To explore the co-expression of *PgMYB* transcripts in Jilin ginseng, we chose to analyze the expression characteristics of these *PgMYB* gene expression data (TPM) of 60 different samples in R language using the WGCNA (Weight Correlation Network Analysis) program package. The soft threshold for constructing the pro joining matrix was set to 0.85, the minimum number of transcripts under the module (a collection of highly inner-connected genes) was set to 30, and the threshold for merging was chosen to be 0.25. Subsequently, the modules’ transcripts were formed into a visual network in Cytoscape V3.9.0 based on their concatenation relationships (Table S5). Finally, we plotted the transcripts in the module in an expression heat map in the R language.

### Response of the *PgMYB* genes to salt stress

The adventitious root materials of Jilin ginseng were treated with different salt concentrations of NaCl (CK, 70 mM, 80 mM, and 90 mM) added to the B5 medium required for their growth. These adventitious root materials were treated with salt stress at 22 °C in the dark for 30 days. Subsequently, all treated adventitious root materials were fast frozen by liquid nitrogen and finally stored in a refrigerator at − 80 °C.

We extracted RNA from salt stress-treated ginseng adventitious roots using the TRIzol method (Biotake, Beijing, China). The cDNA was obtained by reverse transcription using the extracted RNA as a template according to the experimental procedure for reverse transcription provided by HiFiScript gDNA Removal cDNA Synthesis Kit (CWBIO, Beijing, China). cDNA was obtained by reverse transcription according to the experimental protocol provided by UltraSYBR One Step RT-qPCR Kit (Low ROX) (CWBIO, Beijing, China) to perform fluorescent quantitative PCR in ABI 7500 Fast Real-Time PCR System. *Actin 1* gene is an internal reference gene [37]. The 10 μL reaction system consisted of 5 μL UltraSYBR Mixture (Low ROX), 0.2 μL each of right and antisense primers, 1 μL of cDNA template obtained by reverse transcription, and 3.6 μL of ddH_2_O. After three technical and biological replicates, the internal reference gene was normalized, and the expression of the target gene was calculated by the 2^−△△Ct^ method. Finally, bar graphs were plotted by GraphPad prism V8.3.0 and significant differences were calculated between the treated and control groups.

## Results

### Identification of the MYB transcripts in Jilin ginseng

A total of 420 transcripts containing the MYB structural domain were identified in the Jilin ginseng transcriptome database, and these 420 transcripts were generated by alternate splicing of 117 genes. These transcripts were named PgMYB01 - PgMYB117. Table S1 contains the specific information of these transcripts. These include Transcript ID, Gene ID, Transcript Length (bp), DNA sequences (5′ - 3′), ORF (bp/aa), Conserve domain. These nucleic acid sequences range in length from 218 bp–4161 bp, with the shortest fragment length being PgMYB111, the longest fragment length was PgMYB109–01, and their amino acid lengths ranged from 33 aa – 1267 aa.

### Phylogenetic analysis and sequence conservation of the *PgMYB* gene family

**Fig. 1:**
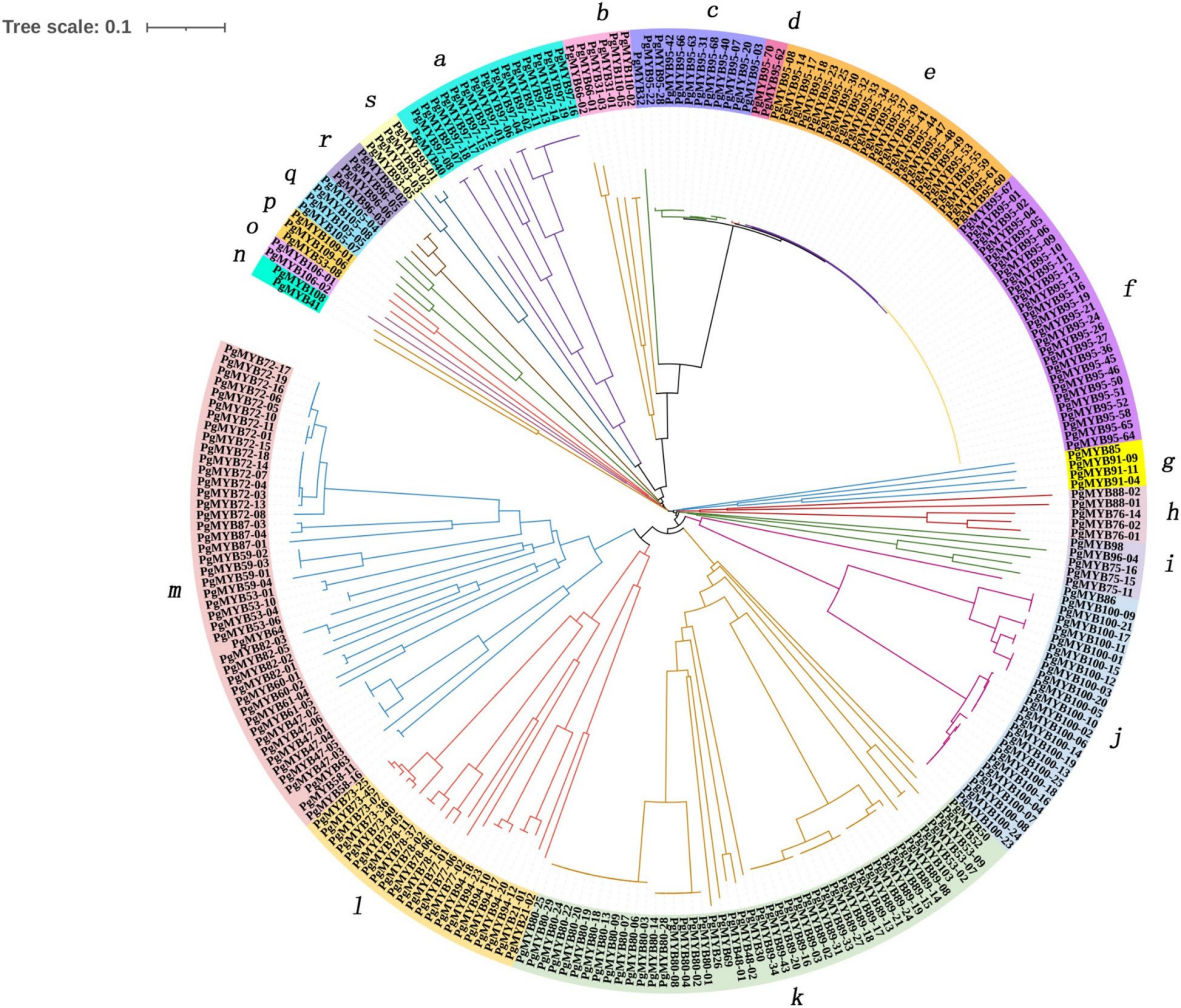
Phylogenetic tree of the PgMYB proteins in *Panax ginseng*. The phylogenetic tree of the *PgMYB* gene family was constructed using 257 *PgMYB* gene. The subfamilies of the *PgMYB* gene family are indicated 19 subfamilies (a to s). The phylogenetic tree was constructed using the Neighbor-Joining (NJ) method with 1000 bootstrap replications

**Fig. 2:**
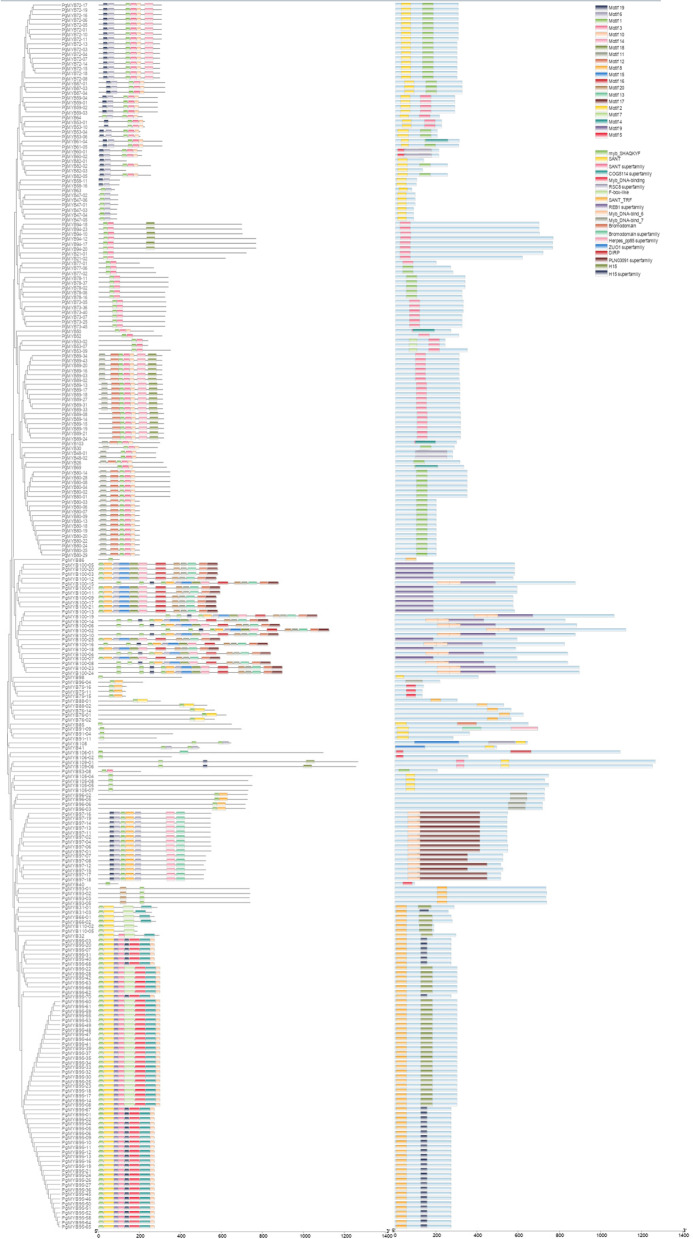
Conserved motif and structural domain analysis of *PgMYB* gene transcripts. Conserved motifs of PgMYB proteins according to their evolutionary relationship. Each motif and structural domains is represented by a colored box. Box length corresponds to the motif and structural domains length

To understand the evolutionary relationships of PgMYB transcripts, we performed a phylogenetic analysis of 257 protein sequences containing MYB structural domains by the NJ method (Fig. 1). These protein sequences were mainly distributed in 19 subfamilies (a - s), among which subfamilies m and k contained 45 and 47 PgMYB transcripts, respectively, and subfamilies n, o and d contained two PgMYB transcripts each. We predicted 20 conserved motifs among these 257 transcripts by MEME analysis. Motif 1, motif 2, motif 6, and motif 8 were identified as conserved motifs of MYB. The structural domain prediction results of these PgMYB protein sequences by NCBI CDD revealed a total of 19 structural domain information, including 7 structural domain information of MYB, in addition to 12 other structural domains (Fig. 2). The different PgMYB transcripts contain different motifs and structural domains in different subclasses, which confers the diversity of *PgMYB* gene functions in ginseng.

### Functional differentiation of the *PgMYB* gene family

**Fig. 3:**
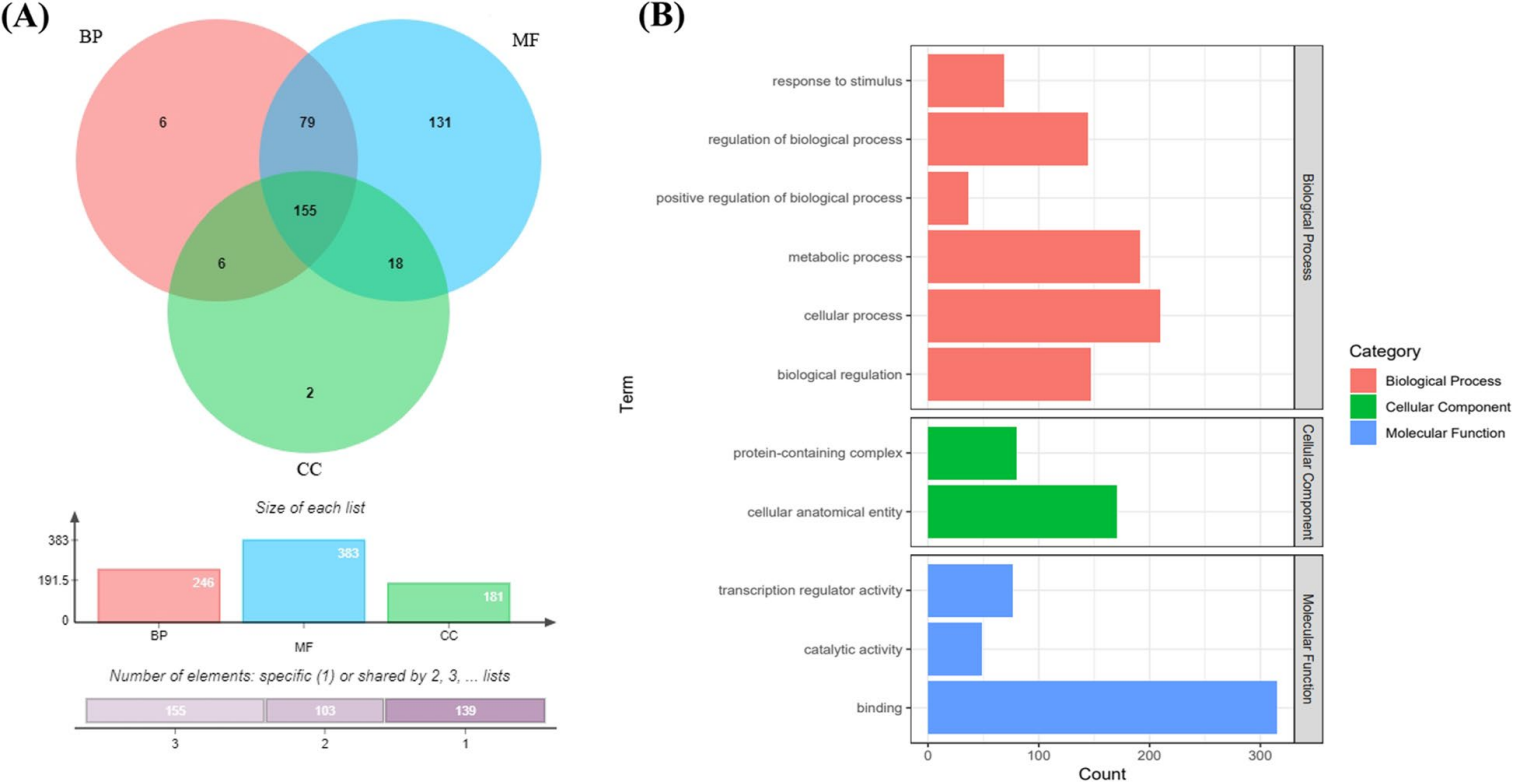
Functional categorization of the *PgMYB* transcripts by gene ontology (GO). **A** Venn diagram of the *PgMYB* transcripts among the biological process (BP), molecular function (MF) and cellular component (CC) functional categories. **B** The *PgMYB* transcripts are classified into 11 functional categories at Level 2, including two CC functional categories (Green), three MF functional category (Blue), and six BP functional categories (Red)

To understand the functions of individual *PgMYB* transcripts, 420 *PgMYB* transcripts were analyzed for GO function annotation (Table S3, Fig. 3). 397 PgMYB transcripts were annotated to any of the three major categories in the GO database (Biological Process (BP), Molecular Function (MF) and Cellular Component (CC)). The remaining 23 *PgMYB* transcripts did not receive annotation information. Of the 397 *PgMYB* transcripts annotated to function, 246 *PgMYB* transcripts were annotated to Biological Process, 181 *PgMYB* transcripts were annotated to Cellular Component, and 383 *PgMYB* transcripts were annotated to Molecular Function. 155 *PgMYB* at the level 2 level, these transcripts were annotated to different nodes under the three major categories, which include MF (binding (315), transcription regulator activity (48), catalytic activity (76)), BP (metabolic process (191), biological regulation (147), cellular process (209), regulation of biological process (144), response to stimulus (68), positive regulation of biological process (36)), CC (cellular, anatomical entity (170), protein entity (170), protein-containing complex (80)). Thus, it can be demonstrated that *PgMYB* transcripts play multiple functions in ginseng.

### *PgMYB* gene duplication and chromosome localization

**Fig. 4:**
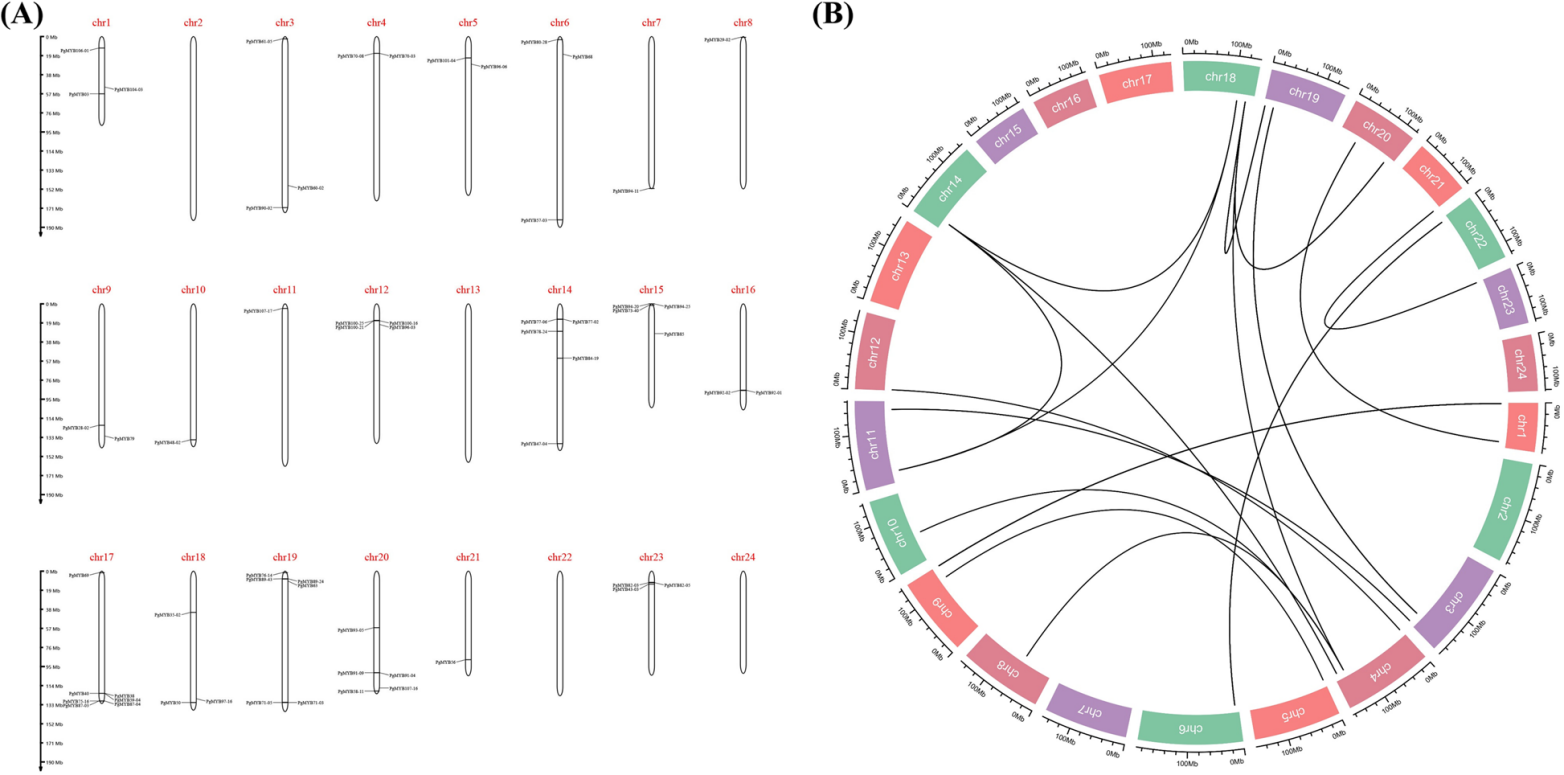
Chromosomal localization and synteny block of the *PgMYB* gene family in *Panax ginseng*. **A** Chromosomal localization of the *MYB* gene family in Jilin ginseng. **B** Synteny block of *PgMYB* transcriptional gene family members within the ginseng genome. Black arcs indicate synteny between genes, Chr: Chromosome, extrachromosomal scale represents the length of chromosome (Mb)

After the local Blast, a total of 138 gene transcripts could be fully matched to the chromosomes of ginseng, and these *PgMYB* transcripts were localized to 20 of the 24 chromosomes. It can be seen from Fig. 4 that chr2, chr13, chr22, and chr24 were not matched to any of the *PgMYB* transcripts. Interestingly, we found that *PgMYB* transcripts were also segmentally duplicated in ginseng by Synteny Block within ginseng. This just proves that the event of whole genome duplication (WGD) is also present in ginseng.

### Expression characteristics of the *PgMYB* transcripts

**Fig. 5:**
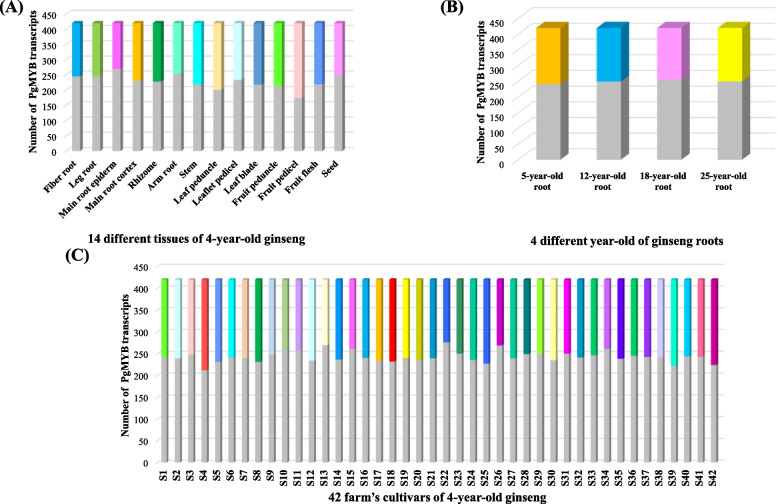
Numbers of the *PgMYB* transcripts expressing across different tissues, year-old of ginseng root, and cultivars. **A** The histogram *of PgMYB* transcripts expressed in 14 different tissues of 4-year-old ginseng. **B** The histogram of *PgMYB* transcripts expressed in 4 different year-old of ginseng roots. **C** The histogram of *PgMYB* transcripts expressed in 42 farm’s cultivars of 4-year-old ginseng

**Fig. 6:**
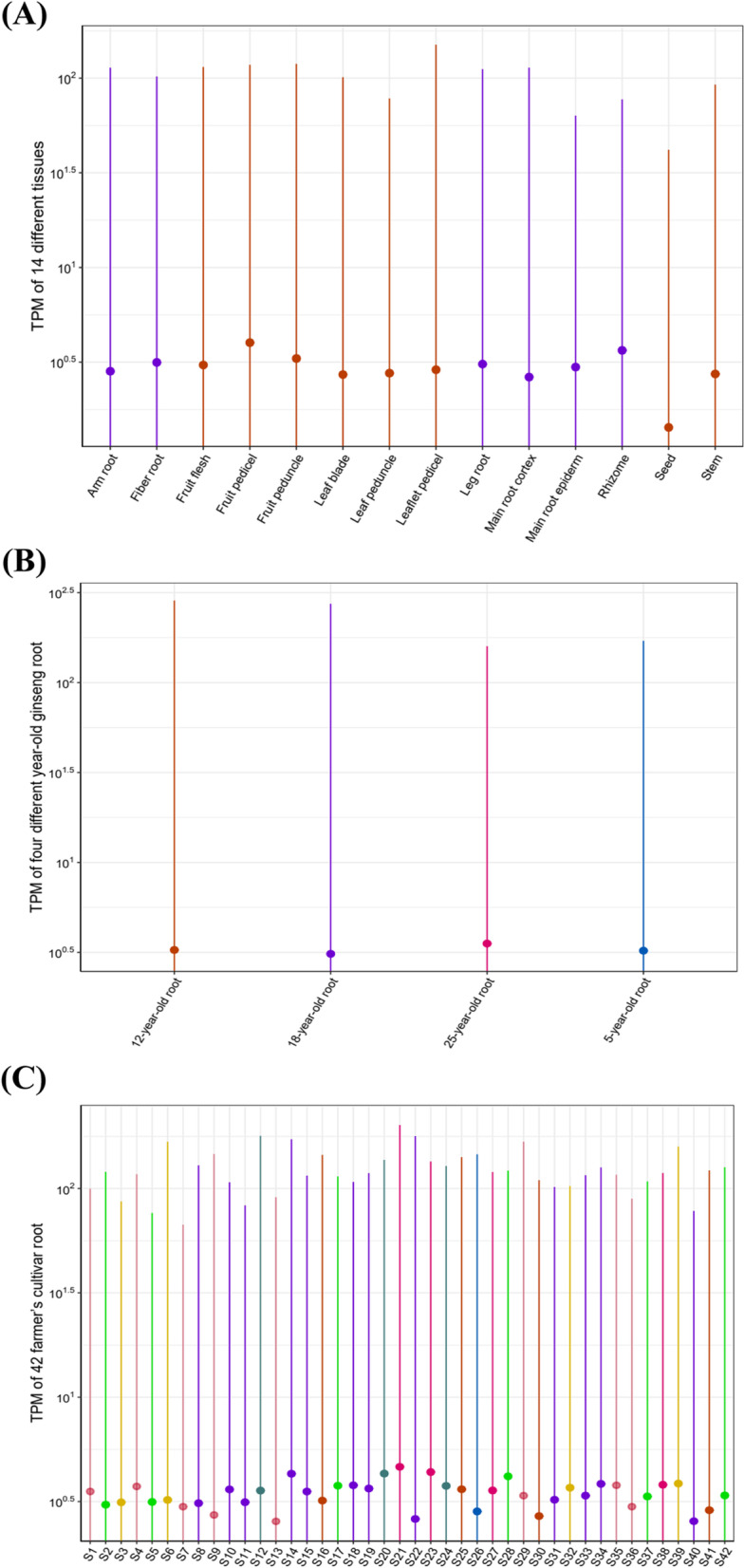
The expression range of *PgMYB* transcripts in Jilin ginseng. **A** The point plot of all *PgMYB* transcripts expression 14 different tissues of 4-year-old ginseng. **B** The point plot of *PgMYB* transcripts expressed in 4 different year-old of ginseng roots. **C** The point plot of *PgMYB* transcripts expressed in 42 farm’s cultivars of 4-year-old ginseng. The point shows the average expression amount of *PgMYB* gene transcripts, and the line shows the expression range of *PgMYB* gene transcripts

To further characterize *PgMYB* transcripts in 14 tissue parts of 4-year-old ginseng (stem, fiber root, fruit peduncle, main root epiderm, fruit pedicel, rhizome, leaf peduncle, arm root, leaflet pedicel, leg root, leaf blade, fruit flesh, main root cortex, and seed), four different age stages of ginseng roots (5, 12, 18, and 25 years), and 42 farm cultivars (S1 - S42), we analyzed the expression of these transcripts (Table S3). Among the 14 tissue sites of four-year-old ginseng (Fig. 5A), 62 *PgMYB* transcripts were expressed in all 14 tissue sites, 62 transcripts were expressed in only one of the 14 tissue sites, and 43 transcripts were not expressed in any of the 14 tissue sites. The number of *PgMYB* transcripts expressed in different tissues showed that *PgMYB* transcripts were more inclined to be expressed in the fruit peduncle of ginseng. From the expression of PgMYB transcripts in ginseng roots at four different age stages (Fig. 5B), 110 *PgMYB* transcripts were expressed at all four age stages, 64 transcripts were expressed at specific periods only and 174 transcripts showed no expression at all four age stages. Among the 42 farm cultivars (Fig. 5C), 48 *PgMYB* transcripts were expressed in all 42 farm cultivars, 17 *PgMYB* transcripts were expressed only in specific farm cultivars, and 79 transcripts did not show expression in any of the 42 farm cultivars. By dotted line plot, we found (Fig. 6) that the average expression of *PgMYB* transcripts was higher in fruit peduncle in 14 tissues. However, in seed, the average expression of *PgMYB* transcripts was lower. In four different-year-old ginseng roots, the average expression of *PgMYB* was higher in 25-year-old roots, and among the 42 farm cultivars, the average expression content of *PgMYB* was lower in two farm cultivars, S13 and S40. The above results suggest that although the same transcript is expressed differently in different tissue parts of four-year-old ginseng, different-year-old ginseng roots in different farming cultivars may be due to the temporal and spatial constraints on the expression of the *PgMYB* transcript.

### Expressions and network analysis of the *PgMYB* transcripts

**Fig. 7:**
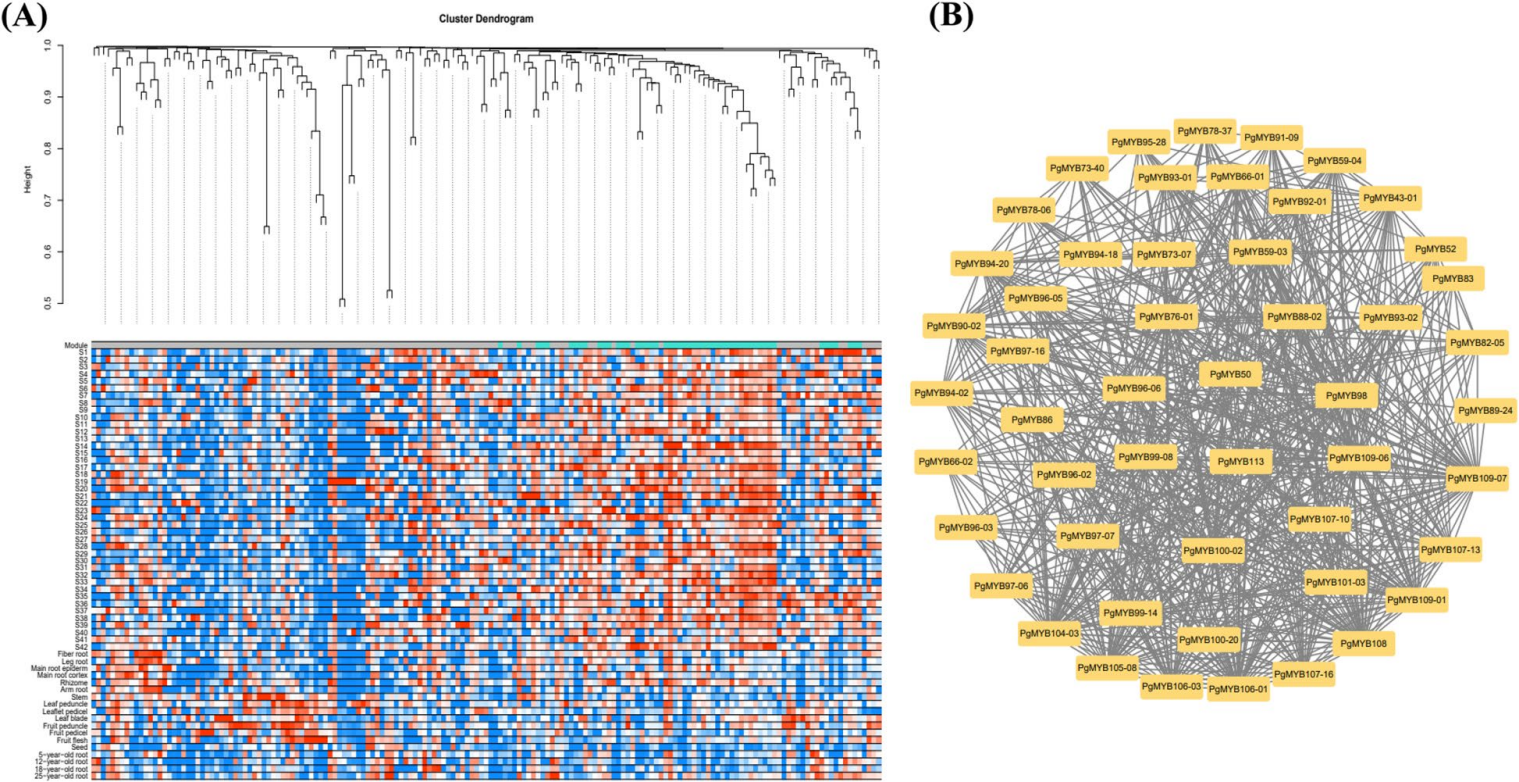
Co-expression network analysis of the *PgMYB* gene transcripts expressed in 60 different samples. **A** Weighted network co-expression analysis of 420 *PgMYB* gene expressions. Turquoise transcripts are assigned the same module, gray transcripts are not assigned to any module as listed. **B** Co-expression network of 51 *PgMYB* gene transcripts under turquoise module

**Fig. 8:**
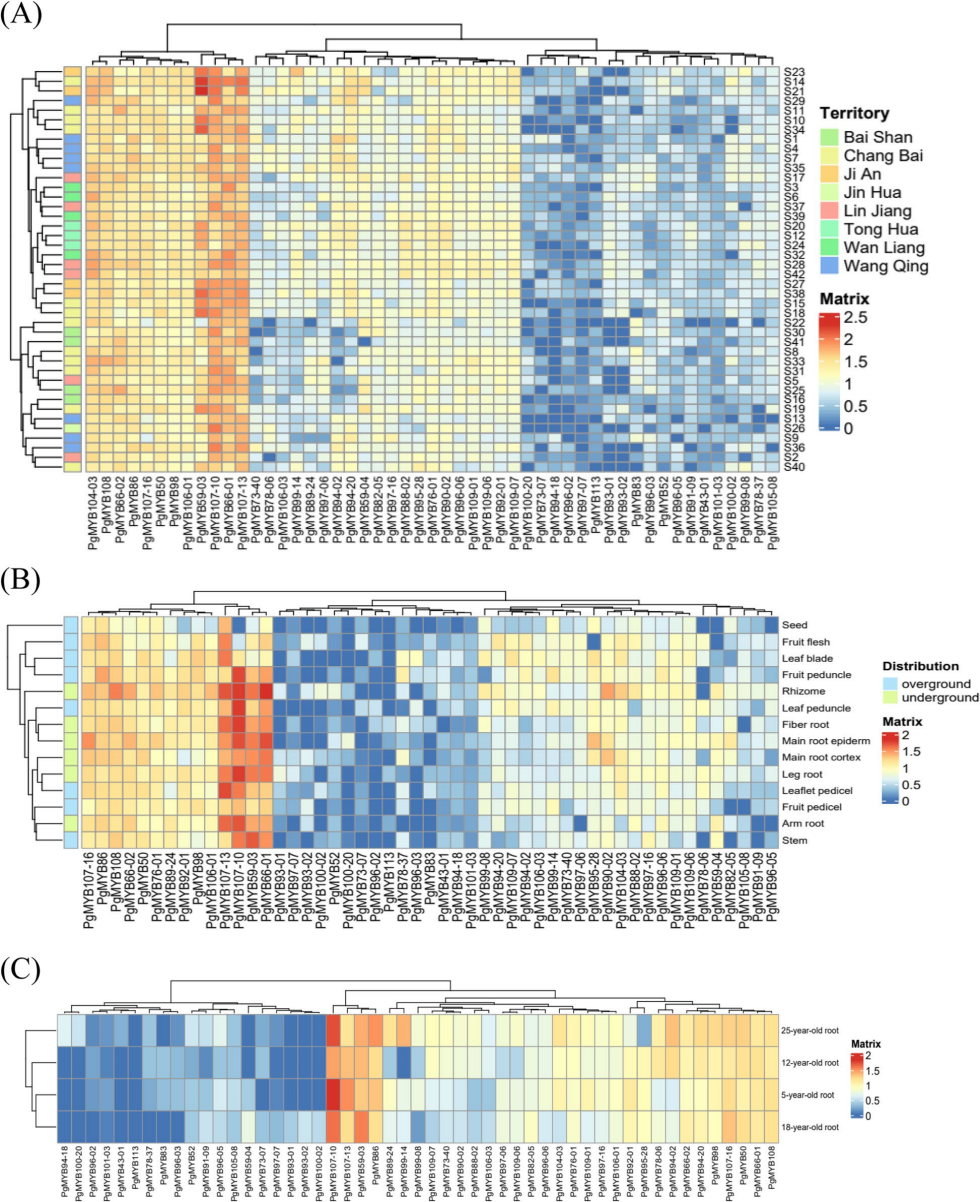
Heatmaps analysis spatiotemporal expression patterns of *PgMYB* transcripts in *Panax ginseng*. **A** The *PgMYB* gene transcripts expressed in the 42 farmer’s cultivars of 4-year-old ginseng roots. **B** The *PgMYB* gene transcripts expressed in the 14 different tissues of 4-year-old ginseng. **C** The *PgMYB* gene transcripts expressed in the 4 different growing years (5, 12, 18, 25 years-old) of ginseng roots

We analyzed the relationship between the expression of these 420 *PgMYB* transcripts using WGCNA. Under the set conditions, we generated one module (turquoise) (Fig. 7A), which contains 51 *PgMYB* transcripts. By visualizing the network results in Cytoscape, we found a tight co-expression relationship between these 51 transcripts (Fig. 7B). Expression pattern analysis of these 51 *PgMYB* transcripts revealed (Fig. 8) that the expression of the same transcript was relatively stable across samples. In 42 farmer’s cultivar roots and 14 different tissues, the expression of *PgMYB59–03*, *PgMYB107–10*, *PgMYB66–01*, and *PgMYB107–13* transcripts were relatively high in four different-year-old ginseng roots, *PgMYB59–03*, *PgMYB107–10*, *PgMYB86*, and *PgMYB107–13* had higher expression content. These transcripts with higher expression levels may have important functions in ginseng.

### Response of the *PgMYB* transcripts to salt stress treatment in ginseng adventitious roots

**Fig. 9:**
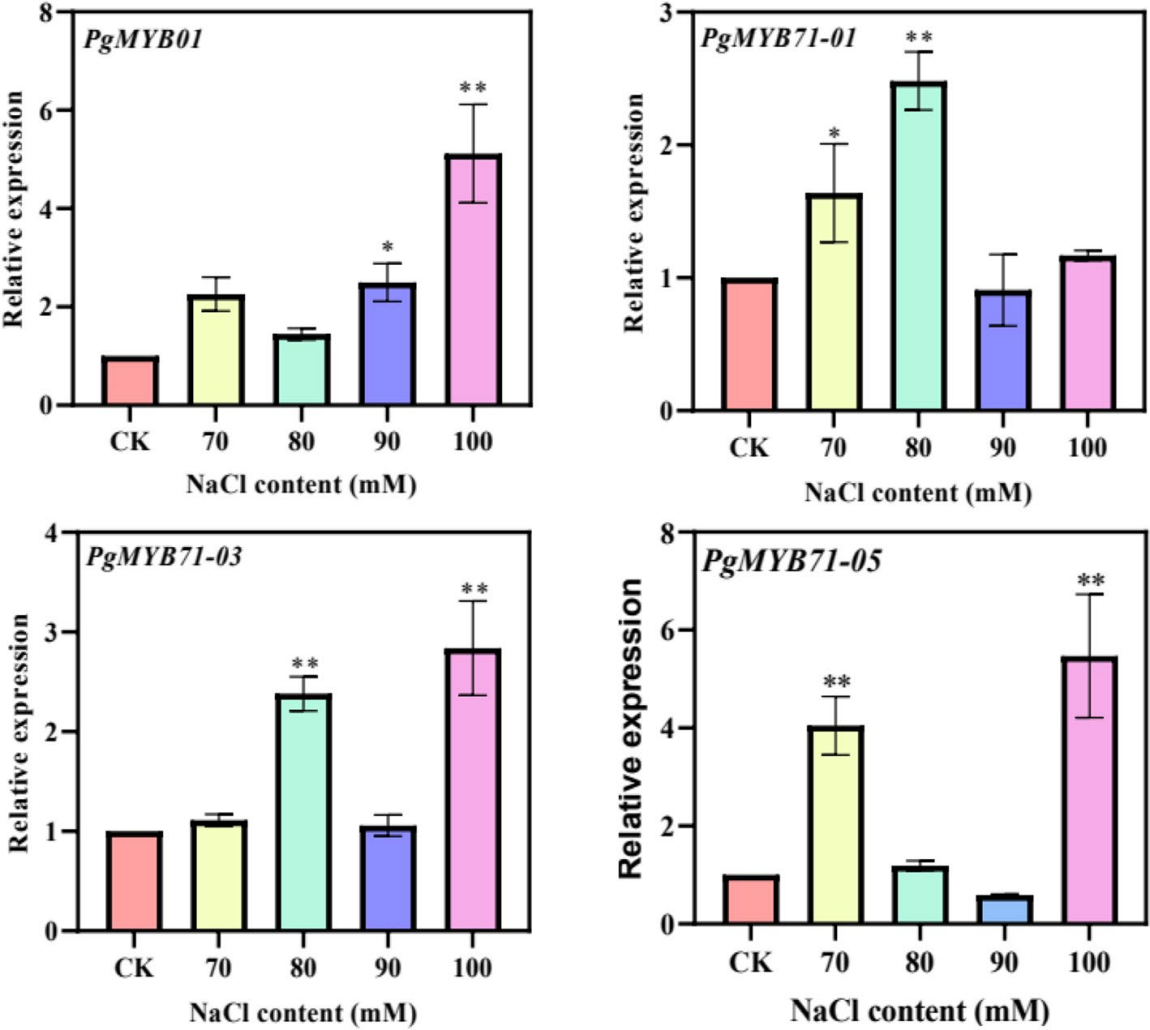
The expressions of *PgMYB01*, *PgMYB71–01*, *PgMYB71–03*, and *PgMYB71–05* gene in the ginseng roots treated with and without salt stresses. The 2^−△△Ct^ method was used to evaluate the relative expression, and the expression levels of genes in the control were defined as “1”. The values are presented as the means of three replicates. “*” as significant at *P ≤ 0.05*, “**” as significant at *P ≤ 0.01*

To investigate the effect of the *MYB* gene family in Jilin ginseng in response to salt stress treatment, we identified some *MYB* gene family members that have been validated to play a role in salt stress treatment, *OsMYB2* [17], *MdMYB4* [38], *OsMYB6* [39], *TaMYB56-B* [40], *LcMYB1* [41], *TaMYB86B* [42], *AtMYB49* [43], the MYB transcription factors (*PgMYB01*, *PgMYB71–01*, *PgMYB71–03*, and *PgMYB71–05*) that may be associated with salt stress were initially screened in ginseng utilizing local Blast. By collating the RT-qPCR results (Fig. 9), we found that the relative expressions of *PgMYB71–01* and *PgMYB71–05* genes were significantly different from the control under the condition of 70 mM NaCl treatment. Under the condition of 80 mM NaCl treatment, the relative expressions of *PgMYB71–01* and *PgMYB71–03* genes were highly significantly different from the control. Only the relative expression of *PgMYB01* gene was significantly different from the control under the condition of 90 mM NaCl treatment. The relative expressions of *PgMYB01* and *PgMYB71–05* genes were 5.1-fold and 5.5-fold higher than the control under 100 mM NaCl treatment conditions, which were extremely significantly different from the control. Among the four transcripts tested in response to salt treatment, *PgMYB01* and *PgMYB71–05* genes were more sensitive to salt stress treatment.

## Discussion

In recent years, ginseng has been mainly used for medicinal functions, which has gradually entered our lives as food and health products [44]. However, the identification and study of *MYB* gene family members in *Panax ginseng* have not been reported. As one of the plants’ largest transcription factor families, MYB transcription factors have been studied in many plants. We screened a total of 117 *PgMYB* genes from the Jilin ginseng transcriptome database that compared with the model plant Arabidopsis (197) and the food crops rice (155), maize (322) [13, 45], and the annual or perennial herb tomato (127) [46]. The number of *MYB* transcription factors in ginseng was relatively small. Alternative splicing produces multiple mRNAs from the same gene through variable splice site selection during pre-mRNA splicing, and these mRNAs encode proteins with different functions [47]. Therefore, the functions of these 420 *PgMYB* transcripts still need to be further verified by relevant experiments. By phylogenetic tree analysis, *PgMYB* transcripts were divided into 19 subfamilies, and there were differences in the number of transcripts between different subfamilies. Conservative motif and structural domain analysis showed that although the *PgMYB* transcripts contain the structural domains and motifs of the *MYB* gene family, the number of these motifs differs somewhat among different subfamilies, thus indicating that the *PgMYB* transcripts may be functionally similar among the same subfamilies.

A large proportion of genes for core biological functions are shared by all eukaryotes [36]. Most MYB proteins function as transcription factors with varying numbers of MYB structural domain repeats, conferring the ability to bind DNA. At the same time, transcription factors further regulate various functional genes under specific developmental and stress conditions [48]. From the results of GO annotation, we found that 315 *PgMYB* transcripts (75%) were annotated to the binding function in molecular function, which also indicates that *PgMYB* mainly plays the function of binding DNA in ginseng. Meanwhile, we noticed that 246 *PgMYB* transcripts (58.6%) were annotated to biological processes, which implies that *PgMYB* transcripts are involved in regulating the growth and developmental processes of Jilin ginseng. This result is consistent with previous studies reported [49]. Interestingly, 181 *PgMYB* transcripts (43.1%) were annotated to cellular components. This is consistent with the report that *MYB103*, *MYB85*, *MYB52*, and *MYB54* in Arabidopsis are necessary for their normal secondary wall synthesis [50].

By analyzing the expression patterns of *PgMYB* in 42 farm cultivars, 14 different tissues, and four different-year-old ginseng roots, we can easily see that although the expression patterns of *PgMYB* transcripts are not consistent, most of them are still expressed in multiple cultivars, tissues, and year-old, which provides a good reference for Jilin ginseng subsequent developmental biology studies, which provides a good reference. At the same time, we also found that a small proportion of transcripts were specifically expressed, and these specifically expressed *PgMYB* transcripts provide favorable candidates for functional studies of ginseng.

Previous studies have reported a hierarchical regulatory network of the *MYB* gene family, and in maize, the transcriptional activator *ZmMYB69* can directly target and activate the expression of *ZmMYB31* and *ZmMYB42*, there by inhibiting lignin biosynthesis in maize [51]. Our co-expression analysis of the weighted network of these 420 *PgMYB* transcripts revealed that a similar hierarchical regulatory network of *PgMYB* transcripts exists in Jilin ginseng, which consists of 51 *PgMYB* transcripts that may be involved in the regulation of secondary metabolites in Jilin ginseng. This result also paves the way for the subsequent study on the interaction mechanism of MYB transcription factors in Jilin ginseng.

A wealth of information has accumulated on the role of proteins in regulating important processes in plants, including development, metabolism, and responses to environmental stresses [52]. An Arabidopsis R2R3-MYB transcription factor, *AtMYB20*, negatively regulates type 2C serine/threonine protein phosphatases to enhance salt resistance [53]. Ectopic expression of a wheat MYB transcription factor gene, *TaMYB73*, improves salt stress resistance in *Arabidopsis thaliana* [54]. In ginseng, a *MYB* gene (*PgMYB1*) was amplified from the hairy roots of ginseng by RT-PCR using a pair of primers corresponding to the conserved sequence of the plant *MYB* gene, which showed a significant up-regulation of expression in 50 mM NaCl treatment for 48 h [21]. This suggests that MYB transcription factors are widely involved in plants’ salt stress resistance process.

## Conclusions

In conclusion, 117 *PgMYB* genes were screened by combining the transcriptome data of Jilin ginseng. The studies on MYB family members in ginseng were enriched by phylogeny, conserved motifs, and structural domains, GO functional annotation, expression patterns and co-expression network analysis. Treatment of ginseng adventitious roots with different concentrations of NaCl revealed that the expression levels of most salt-resistant related *MYB* transcripts were higher under high concentrations of NaCl, thus reducing the harm of salt stress treatment on ginseng adventitious roots. This study provides a data resource for the subsequent functional study of *MYB* gene family in ginseng and provides a genetic reference for salt stress resistance in ginseng.

## Supplementary Information

**Additional file 1: Table S1.** Basic information of *PgMYB* gene family.

**Additional file 2: Table S2.** The PgMYB protein sequences for phylogenetic analysis.

**Additional file 3: Table S3.** The classification, annotation and GO functional categorization of the *PgMYB* gene transcripts.

**Additional file 4: Table S4.** The expressions of the *PgMYB* gene transcripts in 14 tissues, 42 cultivars’ roots and 4 aged roots (TPM).

**Additional file 5: Table S5.** Weight correlation network analysis concatenation relationships and value of *PgMYB* gene transcripts.

## Data Availability

All data analyzed during this study are included in the supplementary information files, and genotypic data have been deposited in the Sequence Read Archive (https://www.ncbi.nlm.nih.gov/sra) to NCBI under BioProject PRJNA302556.
